# Evaluation of intraductal delivery of poly(ethylene glycol)‐doxorubicin conjugate nanocarriers for the treatment of ductal carcinoma in situ (DCIS)‐like lesions in rats

**DOI:** 10.1002/jin2.51

**Published:** 2018-10-15

**Authors:** Zichao Gu, Firas Al‐Zubaydi, Derek Adler, Shike Li, Steven Johnson, Puja Prasad, Jennifer Holloway, Zoltan Szekely, Susan Love, Dayuan Gao, Patrick J. Sinko

**Affiliations:** ^1^ Department of Pharmaceutics, Ernest Mario School of Pharmacy Rutgers, The State University of New Jersey 160 Frelinghuysen Rd. Piscataway New Jersey 08854 USA; ^2^ Rutgers Molecular Imaging Center 41 Gordon Road Suite D Piscataway New Jersey 08854 USA; ^3^ Department of Chemical Engineering Indian Institute of Technology Hauz Khas New Delhi 110016 India; ^4^ Rutgers Cancer Institute of New Jersey 195 Little Albany Street New Brunswick New Jersey 08901 USA; ^5^ DSL Research Foundation Santa Monica California USA

**Keywords:** DCIS rat model, doxorubicin, ductal carcinoma in situ (DCIS), intraductal therapy, mammary gland retention, PEG nanocarrier

## Abstract

Ductal carcinoma in situ is the most commonly diagnosed early stage breast cancer. The efficacy of intraductally delivered poly(ethylene glycol)‐doxorubicin (PEG‐DOX) nanocarriers, composed of one or more DOX conjugated to various PEG polymers, was investigated in an orthotopic ductal carcinoma in situ‐like rat model. In vitro cytotoxicity was evaluated against 13762 Mat B III cells using MTT assay. The orthotopic model was developed by inoculating cancer cells into mammary ducts of female Fischer 344 retired breeder rats. The ductal retention and in vivo antitumour efficacy of two of the six nanocarriers (5 kDa PEG‐DOX and 40 kDa PEG‐(DOX)_4_) were investigated based on in vitro results. Mammary retention of DOX and PEG‐DOX nanocarriers was quantified using in vivo imaging. Histopathologic effects of DOX and PEG‐DOX nanocarriers on mammary ductal structure were also investigated. Cytotoxicities of small linear PEG‐DOX nanocarriers (5 and 10 kDa) were not different from DOX whereas larger PEG‐DOX nanocarriers showed reduced potency. The order of mammary retention was 40 kDa PEG‐(DOX)_4_ > 5 kDa PEG‐DOX >> DOX, in normal and tumour‐bearing rats. Intraductally administered PEG‐DOX nanocarriers and DOX were effective in reducing tumour incidence and increasing survival rate, with no significant differences found among the three treatment groups. However, nanocarriers administered intravenously at the same doses were not effective, and intraductally administered free DOX caused severe local toxicity. Intraductal administration of PEG‐DOX nanocarriers is effective and less toxic than that of free DOX, as well as IV DOX/PEG‐DOX. Furthermore, PEG‐DOX nanocarriers demonstrate the added benefit of prolonging DOX ductal retention, which would necessitate less frequent dosing.

## Introduction

Most breast cancers are initiated in the mammary ducts. Ductal carcinoma in situ (DCIS), the most common type of noninvasive breast cancer, results from the proliferation of malignant epithelial cells within the lumen of the breast duct, generally without penetrating the basement membrane (Lee et al., 2012, Flanagan et al., 2010, Burstein et al., 2004, Peterson et al., 2009, Silverstein, 2000). In rare cases, DCIS with microinvasion has been diagnosed (Pimiento et al., 2011). The incidence of DCIS in the USA was found to have increased drastically from 1975 to 2004 (Virnig et al., 2010), with its rate of occurrence remaining higher than that of any other breast cancer (Lee et al., 2012). This increase in diagnoses is mainly attributed to early detection of the cancer through screening mammography, which detects DCIS as microcalcifications and/or soft‐tissue densities in the breast (Ernster et al., 2002, Lee et al., 2012, Ernster and Barclay, 1997). Although typically considered noninvasive, DCIS has been shown to be associated with increased risk in progression to invasive cancer, based on laboratory and patient data (Dick et al. (n.d.), Leonard and Swain, 2004, Rosai, 1991). In fact, even though DCIS lesions may stay localized for years, 20–50% of untreated DCIS cases eventually progress to invasive breast cancer (Cowell et al., 2013, Sanders et al., 2005). Intervention is therefore vital to preventing the development of invasive breast cancer and/or local recurrence and to providing significant health benefits to patients.

Current treatment regimens involve either lumpectomy plus whole breast radiation therapy or mastectomy (Lee et al., 2012, Leonard and Swain, 2004, Lagios and Silverstein, 1997). To reduce the risk of recurrence, patients may receive adjuvant systemic pharmacotherapy. The initial treatment of DCIS involves surgery. When the entire tumour cannot be excised with clean margins, the breast is completely removed. Although patients can opt for breast‐conserving lumpectomy, this procedure causes the appearance and size of the breast to change. It is the consensus that in most patients with non‐multicentric DCIS, lumpectomy with postoperative breast radiation is just as effective as mastectomy but with a demonstrated decrease in local recurrence (Talamonti, 1996, Soran and Vogel, 1999, Rosai, 1991). Unfortunately, radiation therapy also affects the size and texture of the breast. These cosmetic issues affect the quality of life of the patient, even after reconstructive surgery, thus necessitating the development of a less invasive alternative treatment method for DCIS patients. Chemotherapy is not widely applied for treating DCIS, because of the limited blood supply within the breast duct and the adverse effects resulting from systemic administration. Several studies have reported that within the human breast, there are 5 to 12 non‐anastomosing lactiferous ductal systems that can be approached from openings on the nipple surface (King and Love, 2006). Moreover, there is an increasing realization that most breast cancers arise from ductal epithelial cells (Burstein et al., 2004, Peterson et al., 2009, Silverstein, 2000) and are located in a single lobe of one breast (Tot, 2005b, Tot, 2005a, Tot, 2007, Ohtake et al., 1995). These findings suggest that an intraductal approach for delivering chemotherapeutics provides an alternative opportunity for the local treatment of DCIS, by targeting the carcinoma‐containing lobe (Flanagan et al., 2010, King and Love, 2006).

McFarlin and Gould (2003) were the first to use the intraductal route to infuse retroviral vectors into the mammary glands of rats. Afterward, the feasibility of using intraductal therapy to treat breast cancer was evaluated in several preclinical and clinical studies (Okugawa et al., 2005a, Murata et al., 2006, Mahoney et al., 2009, Chen et al., 2010, Love et al., 2009, Okugawa et al., 2005b). Notably, Okugawa et al. (2005b) treated 1‐methyl‐1‐nitrosourea‐induced tumours intraductally with paclitaxel. They concluded that local administration of paclitaxel might be useful for the treatment of breast cancer. Additionally, Love et al. (2009) conducted a study in women before mastectomy in a phase I clinical trial and demonstrated the minimal toxicity of PEGylated liposomal doxorubicin (DOX) and carboplatin after intraductal administration.

Previously, our group demonstrated that poly(ethylene glycol)s (PEGs) were promising nanocarriers for transpapillary administration and intraductal delivery (Singh et al., 2012a, Gu et al., 2018). The present study continues our previous research by investigating the local pharmacokinetic and pharmacologic effects of intraductally administered PEG‐DOX nanocarriers, in an orthotopic DCIS‐like animal model.

## Materials and Methods

### Materials

Aerrane (isoflurane) was obtained from Baxter Healthcare Corporation (Deerfield, IL). The high‐performance liquid chromatography system, equipped with a Symmetry 300™ C18 column (5.0 μm, 4.6 mm × 50 mm column) as well as ultraviolet and fluorescence detectors, was purchased from Waters (Milford, MA). The In‐Vivo MS FX PRO^®^ system was manufactured by Carestream (Woodbridge, CT) and the M2™ Compact High‐Performance MRI (1T) system from Aspect Imaging (Shoham, Israel). PEG‐DOX nanocarriers were synthesized and characterized according to our previously published method (Gu et al., 2018).

#### Cell culture

The 13762 Mat B III cell line, a rat mammary adenocarcinoma cell line, was obtained from American Type Culture Collection. Cells were maintained in RPMI 1640 medium (Gibco/Invitrogen, Gaithersburg, MD, USA) supplemented with 10% fetal bovine serum, penicillin (200 units/mL), and streptomycin (200 mg/mL) under condition of 5% CO_2_ and 95% humidity at 37°C. Cells with a confluence of less than 90% were used.

#### Animals

Female retired breeder F344 rats were purchased from Hilltop Lab Animals, Inc. (Scottdale, PA). The rats were housed in a room with a 12‐h light/dark cycle, allowed to acclimate for at least a week before the study, and were fed a standard diet and water ad libitum. All animal studies were performed in Association for Assessment and Accreditation of Laboratory Animal Care International accredited animal facilities, under approved protocols from the Rutgers University Animal Use and Care Committee.

### Methods

#### Synthesis and characterization of poly(ethylene glycol)‐doxorubicin nanocarriers

The PEG‐DOX nanocarriers were synthesized as described in our previous paper (Gu et al., 2018). Briefly, commercially available N‐hydroxysuccinimide (NHS)‐activated PEG compounds were mixed with DOX hydrochloride in the presence of diisopropylethylamine in dimethylformamide. The reactions were stirred overnight at room temperature, purified by size exclusion chromatography, and analysed by matrix‐assisted laser desorption ionization time‐of‐flight mass spectrometry. The hydrodynamic radii of the PEG‐DOX nanocarriers were measured at room temperature using dynamic light scattering (Gu et al., 2018).

#### In vitro cytotoxicity of doxorubicin and poly(ethylene glycol)‐doxorubicin nanocarriers

Cell suspensions (3000 cells/0.1 mL/well) were dispensed into 96‐well plates and incubated with PEG‐DOX nanocarriers and DOX for 48 h. Cell viability was evaluated using a CCK‐8 kit (Dojindo, Rockville, MD, USA), by measuring the absorbance at 450 nm with a microplate reader.

#### Orthotopic breast cancer model in F344 rats

Rats were anesthetized with isoflurane and placed under a dissection microscope. The fourth nipple on the left side was cleaned with 70% ethanol. After dilation of the nipple orifice, 13762 Mat B III cells (2.5 × 10^5^/duct) suspended in 0.1 mL serum‐free RPMI 1640 medium were inoculated intraductally, using a 33 G needle attached to a Hamilton syringe (Hamilton, Reno, NV). Tumour dimensions were monitored, and tumour volumes were calculated as follows: evaluated tumour volume = 0.5 × length × width^2^. End point criteria were determined as follows: (1) a maximum tumour volume larger than 10% of the net bodyweight (raw bodyweight minus tumour weight), (2) a mean tumour diameter exceeding 40 mm in rats, (3) ulceration of the tumour, (4) tumour interference with normal movement or function of vital organs (as manifested by signs of distress, such as labored breathing), and (5) inability of the animal to eat or drink. All animals meeting end point criteria were euthanized with CO_2_ and counted as death events.

#### Pharmacokinetics of poly(ethylene glycol)‐doxorubicin and doxorubicin in mammary gland

Retention of DOX and PEG‐DOX nanocarriers was evaluated in mammary glands of both normal and tumour‐bearing rats. In the tumour model, 2.5 × 10^5^ 13762 Mat B III cells were injected into the duct of the fourth mammary gland at day 0, as described previously. PEG‐DOX nanocarriers and DOX (0.83 mg/kg/duct DOX equivalent) prepared in saline were intraductally injected into the same duct on day 2. The mammary contents of the test articles were then measured fluorometrically, via optical imaging.

#### Efficacy studies in F344 tumour model

The F344 female rats were intraductally injected with 13762 Mat B III cells (2.5 × 10^5^ cells/rat). At day 2 after initial cell inoculation, 5 kDa PEG‐DOX, 40 kDa PEG‐(DOX)_4_, and free DOX were injected into the tumour‐bearing rats either intravenously, or intraductally into the same mammary gland as the cell inoculum, at a dose of 0.83 mg/kg DOX equivalent (10 rats per group). The control group received no treatment. Tumour growth and rat bodyweights were monitored. The rats were euthanized via CO_2_ once the tumour size reached the end point criteria. Tumour, liver, lung, spleen, and lymph nodes were excised upon killing and fixed in 10% neutral formalin. The fixed tissue samples were then embedded in paraffin and processed for histological evaluation by routine procedures with hematoxylin and eosin staining.

#### Optical imaging

A day before the start of the study, the rats were shaved with electric clippers and depilated with Veet depilatory lotion. During the experiment, the rats were anesthetized via isoflurane and placed into the imaging method. Whole‐body images were captured by an In‐Vivo MS FX PRO^®^ optical imaging system, at different time points. Excitation/emission wavelengths were set to 480 nm/700 nm, f‐stop was 0.95, and exposure time was 30 sec. Fluorescence intensity of the area injected with PEG‐DOX nanocarriers or free DOX was obtained by subtracting the un‐injected area. The retention half‐lives (*t*_1/2_) were estimated by non‐compartmental pharmacokinetics, using PKSolver [4].

#### Magnetic resonance imaging

Magnetic resonance imaging (MRI) images were acquired using two scans per rat, with one scan covering the lower body starting at the duct and the other covering the upper torso. All scans were run at a 256–250 matrix, with the echo time/repetition time at 80/3787 and a flip angle of 180°. Five averages (with each average improving the image quality) were taken each time. The data were transferred from the MRI to vivoquant (Invicro, Boston, MA, USA), an analytical software program. Within vivoquant, the images were examined for activity (e.g., tumours or fluid, fibrosis or irritation, and metastasis). The images were also compared with the prior scans (self‐control) to compare any noted growth or activity.

#### Preparation of mammary whole‐mount

The rats were euthanized at different days after intraductal inoculation of 13762 Mat B III cells. The mammary glands were excised and fixed in 10% neutral formalin for 24 h. They were then rinsed in distilled water for 15 min. The glands were then dehydrated in serial concentrations of ethanol (70%, 95%, and 100%) for 1 h each and defatted in two changes of xylene for 1 h each. The specimens were thereafter rehydrated and stained with alum carmine. The stained whole‐mounts were dehydrated with ethanol, cleared with xylene, and stored in biopsy pouches filled with methyl salicylate. Whole‐mounts were photographed at a uniform magnification.

#### Histology

Whole‐mount specimens were trimmed and subjected to paraffin embedding. The paraffin blocks were sectioned to 5‐micron thickness and then stained with hematoxylin and eosin.

#### Data analysis

Experimental values were expressed as mean ± standard deviation. Nonlinear regression, survival analysis, and other statistical analyses were conducted using graphpad prism v6 (GraphPad Software Inc., La Jolla, CA, USA). Non‐compartmental pharmacokinetic parameters were calculated using PKSolver [4].

## Results

### Cytotoxicity of poly(ethylene glycol)‐doxorubicin nanocarriers

**Figure 1:**
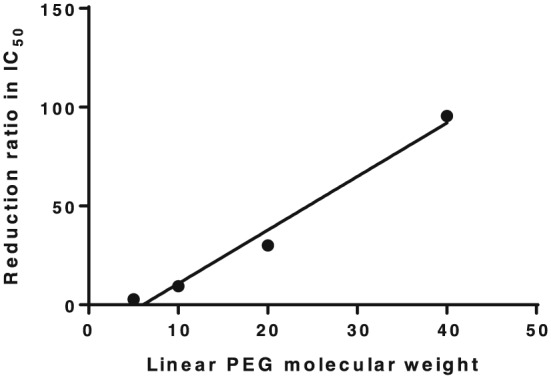
Correlation between the reduction in the ratio (PEG‐DOX vs. DOX) of in vitro IC_50_ against 13762 Mat B III from Table 1 versus linear PEG molecular weight in PEG‐DOX nanocarriers, *R* ^2^ = 0.98. DOX, doxorubicin; PEG, poly(ethylene glycol).

The in vitro cytotoxicity of PEG‐DOX nanocarriers against 13762 Mat B III cells was evaluated, and the data were fit to a sigmoidal nonlinear regression model. The concentrations at which 50% of the cells were viable (IC_50_) were calculated based on the best‐fit model. As shown in Table 1, the IC_50_ value of DOX was 0.025 μM. The IC_50_ values of linear 5, 10, 20, and 40 kDa PEG‐DOX, as well as the 40 kDa PEG‐(DOX)_4_ and 40 kDa PEG‐(DOX)_8_ branched nanocarriers, were 0.071, 0.24, 0.76, 2.42, 0.16, and 0.15 μM, respectively. Among the linear structures of nanocarriers, the 20 and 40 kDa PEG‐DOX were significantly less potent than free DOX and 5 or 10 kDa PEG‐DOX. The ratio of IC_50_ values of linear PEG‐DOX nanocarriers versus DOX displayed a linear correlation to PEG molecular weight (Fig. 1). This finding suggests that PEG size could lower the cellular uptake and release rate of free DOX from nanocarriers by proteolysis, because of steric hindrance. On the other hand, branched nanocarriers demonstrated higher potency than the linear nanocarriers (Table 1). As determined in our previous study (Gu et al., 2018), the hydrodynamic radii were estimated at 3.40 ± 0.16, 3.96 ± 0.23, 4.50 ± 0.15, and 6.34 ± 0.26 nm, for 5, 10, 20, and 40 kDa linear PEG‐DOX nanocarriers.

**Table 1:** Cytotoxicity of PEG‐DOX nanocarriers against 13762 Mat B III cell line.

PEG‐DOX nanocarriers and free DOX	IC_50_ (μM) (95% CI)	IC_50_ ratio (Nanocarriers : DOX)
DOX	0.025 (0.019 to 0.033)	1
5 kDa PEG‐DOX	0.071 (0.056 to 0.089)	2.80
10 kDa PEG‐DOX	0.24 (0.15 to 0.38)	9.40
20 kDa PEG‐DOX	0.76^1^^,^ 2 ^,^ 3 (0.53 to 1.09)	30.04
40 kDa PEG‐DOX	2.42^1^^,^ 2 ^,^ 3 ^,^ 4 (1.07 to 5.49)	95.54
40 kDa PEG‐(DOX)_4_	0.16^4^^,^ 5 (0.13 to 0.20)	6.32
40 kDa PEG‐(DOX)_8_	0.15^4^^,^ 5 (0.11 to 0.21)	5.96

DOX, doxorubicin; PEG, poly(ethylene glycol).

Cytotoxicity studies were conducted as described in Materials and Methods. One‐way analysis of variance with Tukey multiple comparison test was performed.

1 *P* < 0.05 versus DOX.

2 *P* < 0.05 versus 5 kDa PEG‐DOX.

3 *P* < 0.05 versus 10 kDa PEG‐DOX.

4 *P* < 0.05 versus 20 kDa PEG‐DOX.

5 *P* < 0.05 versus 40 kDa PEG‐DOX.

### Orthotopic breast cancer model by intraductal inoculation

**Figure 2:**
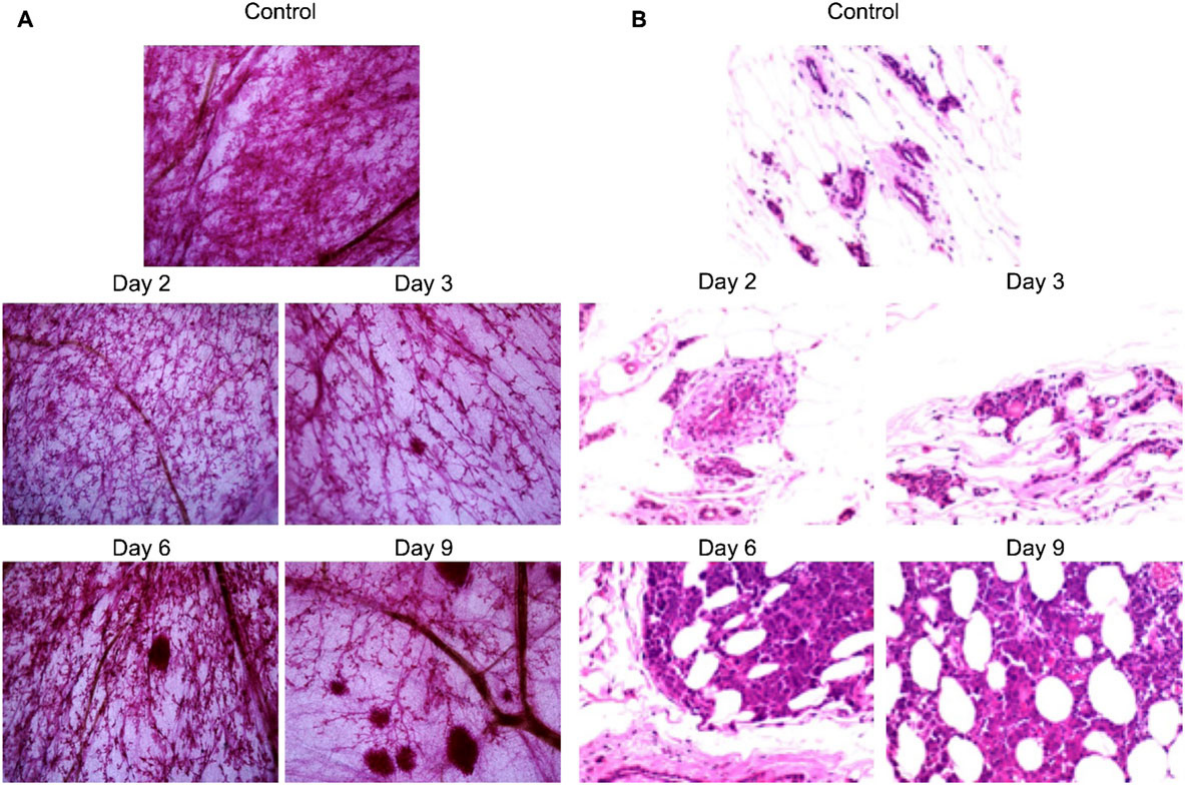
Tumourigenicity of 13762 Mat B III cells inoculated intraductally in F344 rats. The rats were injected with 2.5 × 105 cells/duct and thereafter were killed at the indicated time points. (A) Representative microscopic images of mammary whole‐mounts. The images were acquired with 2× objective. (B) Hematoxylin and eosin staining of paraffin sections of corresponding mammary samples from F344 rats. Microscopic images were taken with a 40× objective.

To mimic the clinical observation that most breast cancer is initiated and developed inside the ducts, 13762 Mat B III cells were directly inoculated into the primary ducts of F344 rats. The rats were injected with 2.5 × 10^5^ cells and euthanized at days 2, 3, 6, and 9 to examine tumour growth at the early stage of tumourigenesis. The cell take rate by F344 rats was 100%. The mammary whole‐mount images are shown in Figure 1. No noticeable microtumours were observed under low power microscopic magnification at day 2 in mammary whole‐mounts (Fig. 2(A)). Starting at day 3, microtumours became visible and enlarged over time (Fig. 2(A)).

The histopathological examination revealed frequent alteration with mitosis in the ductal epithelial cells of the inoculated mammary ducts and the development of DCIS‐like lesions by day 2 after cell inoculation (Fig. 2(B)). Beginning on day 3, tumour invasion into the mammary fat pad was observed.

Various cell loads of 13762 Mat B III were also tested, and tumour growth consistently displayed cell load‐dependent and time‐dependent characteristics throughout all studies (data not shown). A cell load of 2.5 × 10^5^ appeared to provide a favorable tumour growth time‐course for performing the efficacy studies. Additionally, most rats developed metastasis to ipsilateral and contralateral lymph nodes (data not shown). Thus, intraductal inoculation of 13762 Mat B III cells was demonstrated to be a valid breast cancer model mimicking DCIS‐like lesions and progression of DCIS to invasive breast cancer, for further efficacy studies.

### Mammary gland retention of poly(ethylene glycol)‐doxorubicin nanocarriers in normal and tumour carrying F344 rats

**Figure 3:**
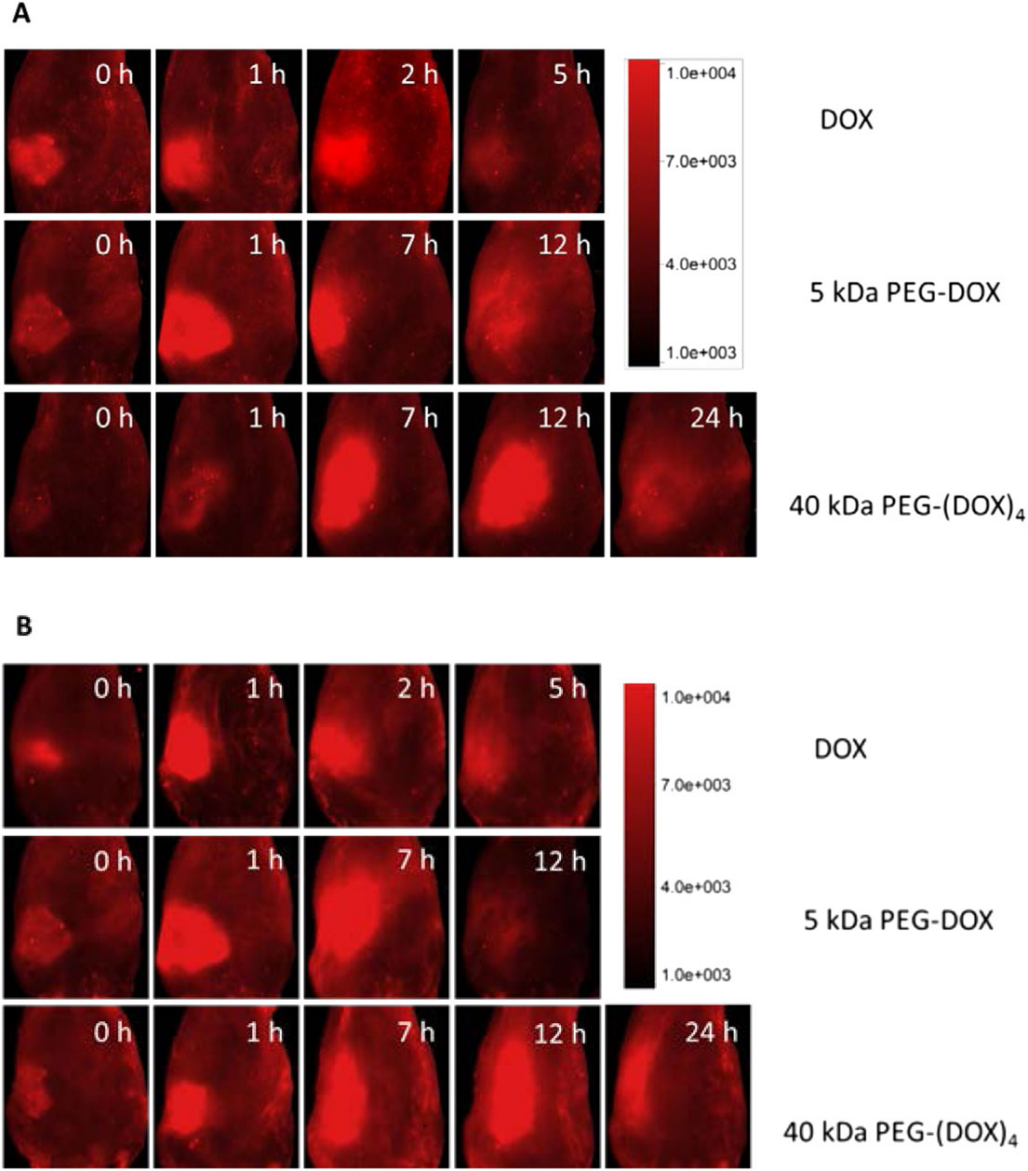
Optical imaging of DOX and PEG‐DOX nanocarriers dosed intraductally into mammary glands of F344 rats. The samples were administered into the mammary ducts of the rats and then imaged at different time points using an In‐Vivo MS FX PRO^®^ optical imaging system. (A) F344 normal rats; (B) F344 rat tumour model at day 2 after tumour cell inoculation (*n* = 3). No difference in mammary retention was observed between (A) normal and (B) tumour‐bearing rats (Fig. 4 and Table 2). DOX, doxorubicin; PEG, poly(ethylene glycol).

**Figure 4:**
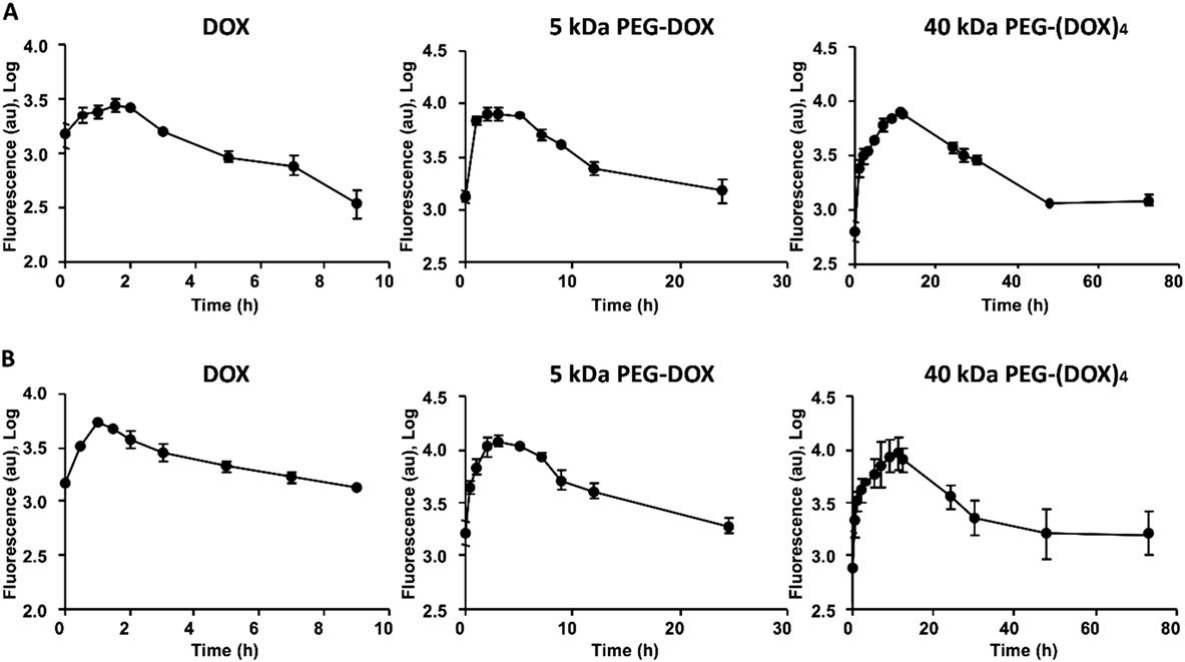
Mammary retention of DOX and PEG‐DOX nanocarriers in F344 rats. DOX and PEG‐DOX nanocarriers were administered intraductally into (A) normal and (B) tumour‐bearing rats. Fluorescence intensities were measured with an optical imager over time, as shown in Figure 3. Each point represents mean ± standard deviation (*n* = 3). DOX, doxorubicin; PEG, poly(ethylene glycol).

The PEG‐DOX nanocarriers and DOX were administered intraductally into normal and tumour‐bearing mammary ducts of F344 rats. Whole‐body fluorescence images were captured with an optical imager. Fluorescence intensities of free DOX, 5 kDa PEG‐DOX, and 40 kDa PEG‐(DOX)_4_ nanocarriers in the mammary gland exhibited a rapid increase and subsequent reduction with time, which was consistent in both normal and tumour‐bearing rats (Figs 3, 4).

**Figure 5:**
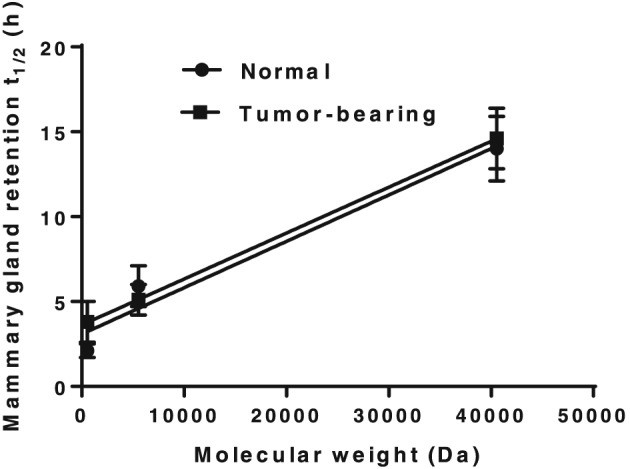
Correlation between poly(ethylene glycol)‐doxorubicin nanocarrier molecular weight and mammary retention half‐life. *R* ^2^ = 0.95 and 1.00 in normal and tumour‐bearing rats, respectively.

The mammary gland retention half‐life (*t*_1/2_), defined as the time required for fluorescence intensity to reach 50% of its peak value, was estimated by non‐compartmental pharmacokinetic model (Table 2). In normal rats, the *t*_1/2_ of free DOX was 2.1 ± 0.4 h, whereas those of the 5 kDa PEG‐DOX and 40 kDa PEG‐(DOX)_4_ were 5.9 ± 1.2 and 14.0 ± 1.9, respectively. Conversely, in tumour‐bearing rats, the *t*_1/2_ of free DOX, 5 kDa PEG‐DOX, and 40 kDa PEG‐(DOX)_4_ were 3.8 ± 1.2, 5.1 ± 0.9, and 14.6 ± 1.8 h, respectively. The nanocarriers exhibited significantly longer mammary retention than the free DOX in both normal rats and tumour‐bearing groups. Under both normal and tumour conditions, 40 kDa PEG‐(DOX)_4_ manifested the longest retention time. On the other hand, all test articles showed similar mammary retention between the normal and tumour groups (Table 2). The results demonstrated that PEG‐DOX nanocarriers were retained for a longer period in the mammary gland and that the retention was not affected by the presence of ductal tumours. Furthermore, there exists a strong correlation between ductal retention half‐life and nanocarrier molecular weight, in both normal and tumour‐carrying rats (Fig. 5).

**Table 2:** Half‐lives (*t*_1/2_) of PEG‐DOX nanocarriers in normal and tumour‐bearing mammary glands of F344 rats.

PEG‐DOX nanocarriers^1^^,^ 2 ^,^ 3	*t*_1/2_ (h)^1^^,^ 2 ^,^ 3	
	Normal rats	Tumour‐bearing rats
DOX	2.1 ± 0.4	3.8 ± 1.2
5 kDa PEG‐DOX	5.9 ± 1.2^4^	5.1 ± 0.9
40 kDa PEG‐(DOX)_4_	14.0 ± 1.9^4^^,^ 5	14.6 ± 1.8^4^^,^ 5

DOX, doxorubicin; PEG, poly(ethylene glycol).

1 *t*_1/2_ is defined as time at which fluorescence reaches 50% of its peak value.

2 *t*_1/2_ = ln2/*k*_el_, where *k*_el_ is the elimination rate constant.

3 *k*_el_ is calculated by regression of semi‐logarithmic fluorescence versus time as lnF = lnb − *tk*_el_.

4 *P* < 0.05, versus DOX in corresponding groups (*n* = 3).

5 *P* < 0.05, versus 5 kDa PEG‐DOX in corresponding groups (*n* = 3). Two‐way analysis of variance with Bonferroni multiple comparison test was performed.

### Antitumour efficacy of poly(ethylene glycol)‐doxorubicin nanocarriers in F344 tumour model

**Figure 6:**
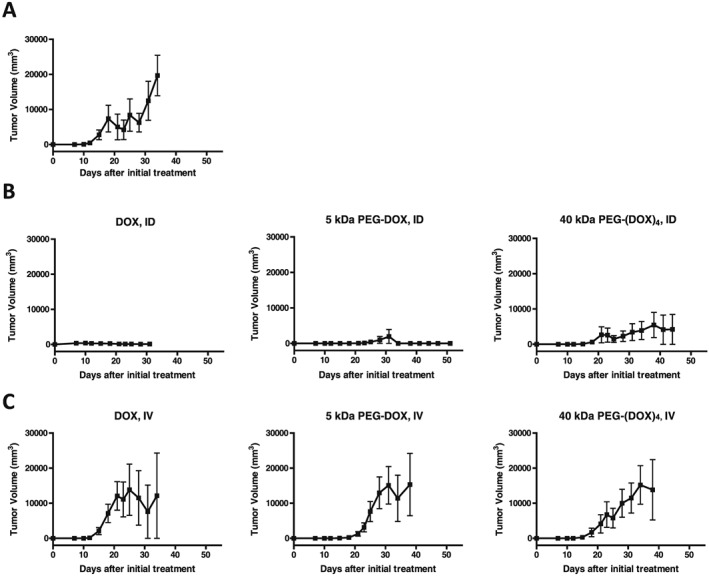
Effect of PEG‐DOX nanocarriers administered by different routes on tumour growth in orthotopic breast cancer model. F344 retired female breeder rats were inoculated with 13762 Mat B III cells (2.5 × 105 cells/rat) into the fourth mammary duct. Two days later, the rats received either intraductal (ID) treatment into the same duct or intravenous (IV) treatment with DOX or PEG‐DOX nanocarriers at doses of 0.83 mg/kg DOX equivalents. (A) Control group, rats received no treatment; (B) rats received intraductal treatment; (C) rats received intravenous treatment. Each point represents the mean ± standard deviation (*n* = 3–9). DOX, doxorubicin; PEG, poly(ethylene glycol).

**Figure 7:**
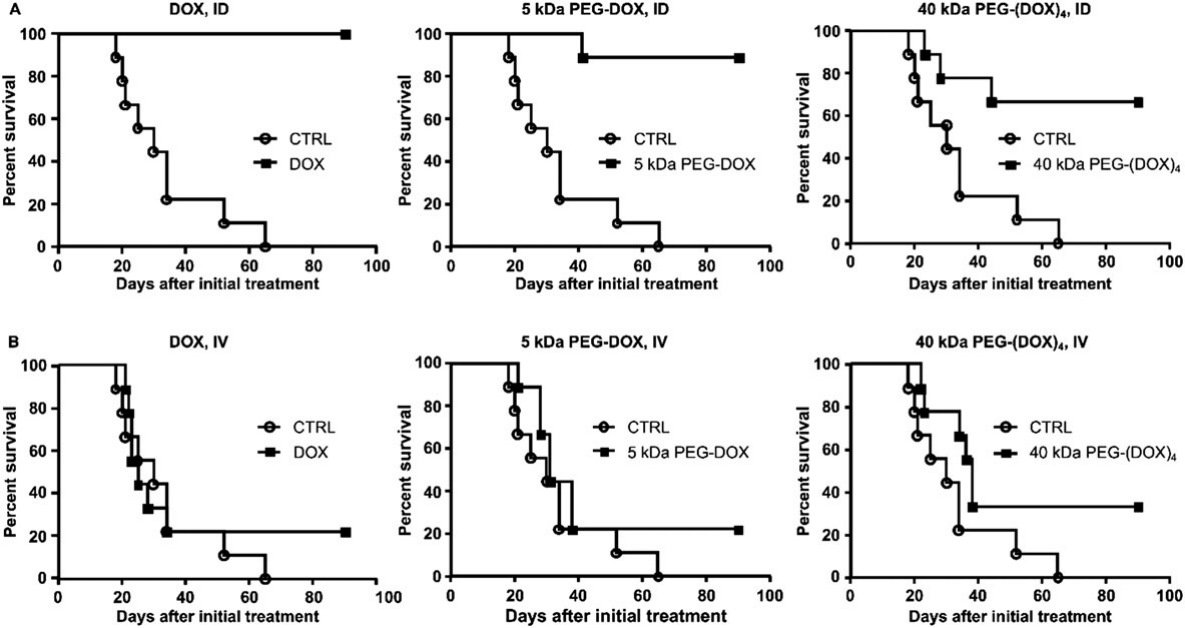
Intraductal treatment with DOX and PEG‐DOX nanocarriers increased survival in the orthotopic breast cancer model. (A) Rats treated with intraductal therapy (ID); (B) rats treated with intravenous therapy (IV). There is insignificant difference among all ID‐treated groups (A). DOX, doxorubicin; PEG, poly(ethylene glycol).

The effect of PEG‐DOX nanocarriers was investigated using the orthotopic breast cancer model. Free DOX and PEG‐DOX nanocarriers were injected either intraductally or intravenously, at day 2 post‐tumour cell inoculation into the mammary duct. Tumour growth, represented as tumour volume, is shown in Figure 6. All intraductal treatments exhibited none to modest tumour growth, compared with the control group (Fig. 6(B)). Intravenous treatments given at the same dose showed greater tumour growth than that of the intraductal treatments (Fig. 6(C)). Mandatory euthanasia of rats meeting end point criteria during the study caused large variability in the results; thus, it was not possible to conduct a reasonable statistical analysis using tumour volume as a marker of efficacy. No significant bodyweight loss was observed during the treatment (data not shown). In the present study, all humane end point euthanasias were performed because of tumour size reaching end point criteria. These losses were counted as death events. Survival curves in Figure 7 were generated from an efficacy study. Statistical difference between survival curves was assessed using a Gehan–Breslow–Wilcoxon test. In all intraductal treatment groups, free DOX, 5 kDa PEG‐DOX, and 40 kDa PEG‐(DOX)_4_ exhibited remarkable significance in survival percentage at levels of *P* < 0.0001, *P* = 0.0002, and *P* = 0.0117, respectively, as compared with the untreated control group (Fig. 7(A)). No significant difference was found among the three intraductally treated groups. In contrast, the intravenously treated groups did not display statistical significance at the same dose level compared with the control group (Fig. 7(B)). After 90 days of treatment, the percentage of tumour‐free rats in each group was as follows: 100% in the free DOX group, 88.9% in the 5 kDa PEG‐DOX group, and 66.7% in the 40 kDa PEG‐(DOX)_4_ group. The intraductal treatment groups had higher survival rates than their corresponding intravenously treated groups, which were 22.2%, 22.2%, and 33.3% in DOX, 5 kDa PEG‐DOX, and 40 kDa PEG‐(DOX)_4_ groups, respectively. These results demonstrate that the intraductal approach is more effective than IV in treating intraductal mammary tumours.

### Doxorubicin, not poly(ethylene glycol)‐doxorubicin nanocarriers, affected the mammary ductal structure

**Figure 8:**
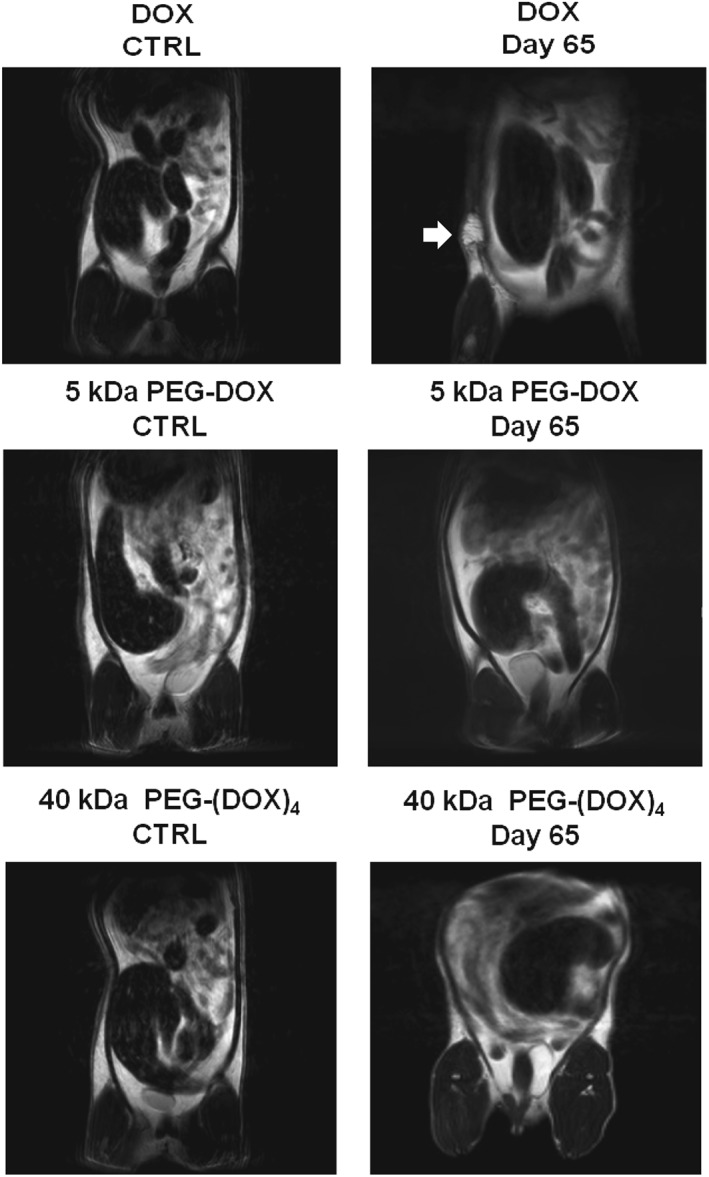
Magnetic resonance imaging images of F344 rat mammary glands treated with DOX and PEG‐DOX nanocarriers. The rats were treated with 0.83 mg/kg/duct of DOX and were scanned for magnetic resonance imaging images after 65 days. Soft lumps were observed in mammary glands (arrowhead) of DOX‐treated rats but not in those receiving PEG‐DOX nanocarriers. The soft lumps were considered to be due to local toxicity of intraductal administration of free DOX. DOX, doxorubicin; PEG, poly(ethylene glycol).

Magnetic resonance imaging was performed to monitor mammary tumour development upon intraductal administration of PEG‐DOX nanocarriers and free DOX. In free DOX‐treated rats, we found that soft lumps developed in both normal and tumour‐bearing rats (Fig. 8). The soft lumps persisted in the mammary gland for more than 2 months (until the termination of the study), whereas no lumps were detected in the PEG‐DOX nanocarrier‐treated groups (Fig. 8).

**Figure 9:**
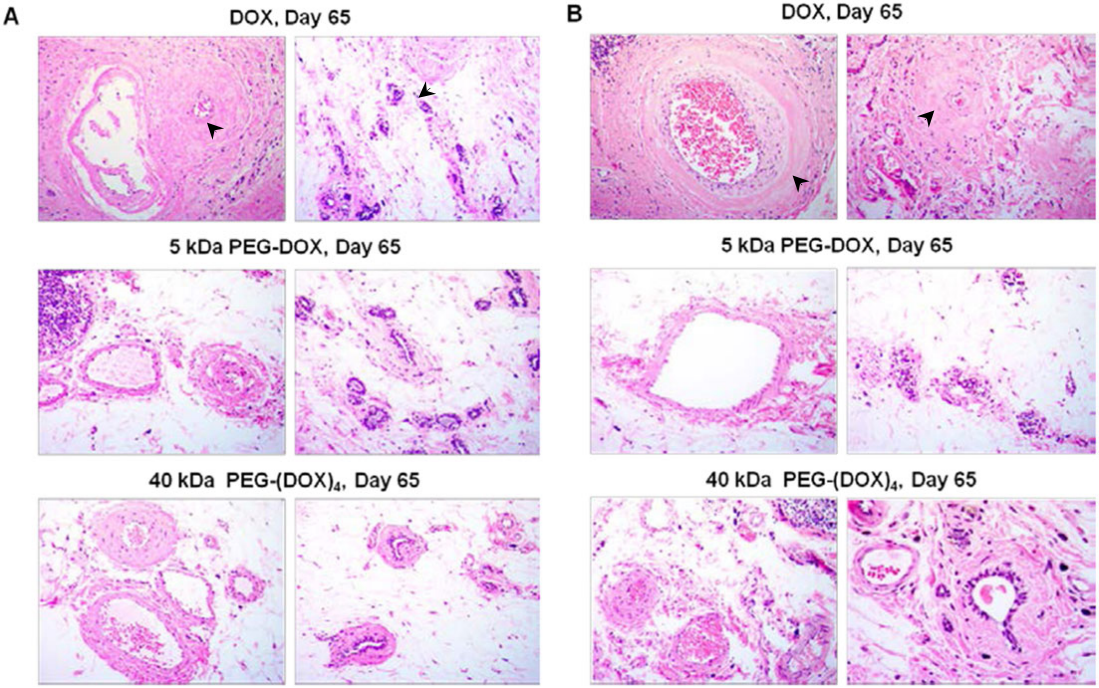
Histology of F344 rat mammary ducts treated with DOX and PEG‐DOX nanocarriers. DOX or PEG‐DOX nanocarriers were intraductally administered into normal and tumour‐bearing rats. The animals were euthanized at 65 days posttreatment. Paraffin sections were stained with hematoxylin and eosin. (A) Normal rats treated with DOX or PEG‐DOX nanocarriers; (B) tumour‐bearing rats treated with DOX or PEG‐DOX nanocarriers. Arrows indicate morphological changes in the sections. Microscopic images were taken at 40× objective. DOX, doxorubicin; PEG, poly(ethylene glycol).

Figure 9 displays hematoxylin and eosin stained mammary glands sampled from normal and tumour‐bearing rats treated with DOX and PEG‐DOX nanocarriers, 65 days after treatment. Increased thickness of small arteries and veins, as well as shrunken mammary gland ducts (arrows), was noted in DOX‐treated rats. On the other hand, PEG‐DOX nanocarrier‐treated ducts displayed normal morphology of arteries, veins, and mammary ducts. These results indicate that DOX altered mammary ductal structure while PEG‐DOX nanocarriers did not, implying that PEG‐DOX nanocarriers are less toxic than free DOX.

## Discussion

Even though DCIS lesions may stay localized for years, 50% of untreated DCIS cases eventually progress to invasive breast cancer. The intraductal drug delivery approach to treating DCIS is an attractive opportunity to enhance local exposure while limiting systemic toxicity, as well as reducing the need for surgery and/or radiation. In addition to several animal studies, a recent human study has showed the effectiveness and potential of intraductal delivery for curing localized breast cancer. While this is promising, further investigation into the characteristics of the mammary duct (e.g., permeability and microenvironment) is critical to improve the efficacy of ductal therapy.

The present study demonstrated that PEG‐DOX nanocarriers possess antitumour potency via mammary ductal administration in an orthotopic rat model of breast cancer. In breast cancer treatment, the recommended dosage for DOX is 60 to 75 mg/m^2^ every 21 days, with the cumulative dose not exceeding 550 mg/m^2^ by intravenous route, in the form of hydrochloride salt (Hande, 1998, Fornari et al., 1994). Because systemic administration of free DOX always causes adverse effects (e.g., cardiotoxicity (Prados et al., 2012)), a local and relatively low dose would minimize systemic exposure and provide a good therapeutic option for DCIS breast cancer treatment. In the present study, a singly administrated dose of 0.83 mg/kg represented 1/12 of the equivalent DOX dose of 60 mg/m^2^. Moreover, the dose was delivered directly to the mammary duct, plausibly leading to a higher local drug concentration in the affected area than systemic administration could ever achieve and resulting in more potent killing of the cancerous cells in the mammary duct. This was evidenced by the superior therapeutic outcome displayed by the intraductally treated groups (Fig. 7(A)). However, intravenous treatment given at the same dose did not show a significant therapeutic effect (Fig. 7(B)).

All PEG‐DOX nanocarriers with various molecular weights and architectures exhibited cytotoxicity in vitro against 13762 Mat B III, a rat breast cancer cell line. The potency of small PEG nanocarriers (5 and 10 kDa) was not different from that of free DOX, while larger PEG‐DOX (20 and 40 kDa) showed reduced potency. Surprisingly, branched PEG‐DOX nanocarriers were more effective than their linear counterparts. This trend was consistent with our prior findings using MCF7, a human breast cancer cell line (Gu et al., 2018). We previously reported that the hydrodynamic radii of PEG or PEG‐DOX nanocarriers increased with increasing molecular weights of PEG nanocarriers (Singh et al., 2012a, Singh et al., 2012b, Gu et al., 2018). Specifically, a larger linear PEG moiety led to lower cellular uptake and limits the accessibility of amide linkage to the hydrolytic action of proteases because of higher steric hindrance (Banerjee et al., 2012). Thus, the reduction in cytotoxicity of PEG‐DOX nanocarriers in comparison with the free DOX was speculated mainly because of low cellular uptake associated with PEG conjugation and consequent reduced accessibility of proteases to the amide linker (Ye et al., 2015). The multiple arm handiness and limited molecular flexibility of branched PEG in comparison with linear PEG resulted in improved in vitro biological activity (Zhao et al., 2008).

Based on the cytotoxicity results, linear 5 kDa and four‐arm 40 kDa PEG‐DOX nanocarriers were selected for ductal retention and efficacy studies. In our previous studies, the influence of molecular size and structure of PEG on PEG‐DOX nanocarrier retention in mammary ducts of Sprague–Dawley (SD) rats was investigated. Because F344 retired breeders were used instead of SD rats to establish a breast cancer tumour model, the influence of molecular size of nanocarriers on the ductal retention in normal and tumour‐bearing F344 rats was reassessed. Because of the larger molecular size and inherent viscosity and/or aggregate formation of their aqueous solution (4 and 8% *w*/*v*), PEG‐DOX nanocarriers exhibited prolonged ductal retention half‐lives with respect to free DOX, which was consistent with the results we obtained previously in SD rats (Veronese et al., 2005). No differences in data trends were noted between normal F344 rats and F344 tumour‐bearing animals. The ductal tumour lesions may not have been extensive enough to affect the epithelial penetration of either DOX or PEG‐DOX nanocarriers.

The major aim of this study was to evaluate the chemotherapeutic effects of PEG‐DOX nanocarriers using an intraductal approach. By using a well‐characterized F344 rat tumour model, the current results show that survival rates in the intraductal therapy groups were significantly improved, and tumour volumes were smaller in comparison with the intravenous groups. Meanwhile, it is interesting to note that no evidence of tumours was found in the intraductal groups treated with free DOX, yet soft lumps in mammary glands were palpable after 3 days post‐intraductal injection in both normal F344 and F344 tumour‐bearing rats. Pathology studies revealed increased thickness of small arteries, veins, and the fibroblast stroma layer in the DOX‐treated mammary glands. This suggests that free DOX affects ductal structure, whereas no lumps were found in the intraductal groups treated with PEG‐DOX nanocarriers. The minimal ductal toxicity exhibited by the nanocarriers was due to (1) nominal proteolytic activity in normal tissues as compared with cancerous tissue (Anderson and Cui, 2017), (2) low cellular uptake of nanocarriers (Banerjee et al., 2012), and (3) high viscosity of the nanocarrier solution, effectively slowing the release of the nanocarriers.

Here we have presented evidence that intraductal delivery of PEG‐DOX nanocarriers is effective for treating mammary tumours developed in the F344 tumour model, with little systemic exposure. In the future, this approach may allow the use of many promising anticancer agents whose clinical application is limited because of systemic toxicity issues.

## Conclusions

In summary, this work presented preclinical data supporting the effectiveness of intraductal delivery of PEG‐DOX (linear 5 kDa and four‐arm 40 kDa) nanocarriers in treating mammary tumours in the F344 rat tumour model. Unlike free DOX, no local inflammation or alteration of ductal structure was observed in the intraductal groups treated with PEGylated DOX nanocarriers, suggesting a reduced local toxicity. In addition, we have noninvasively assessed the retention of intraductally administered nanocarriers in both normal F344 rats and the F344 tumour model. There was no significant difference of retention between normal F344 animals and the F344 tumour model. The data presented here will aid in the design of further drug delivery systems for intraductal therapy, to treat DCIS in humans.

## Funding

This work was supported by the grant from National Institutes of Health HIT IT (R01AI084137‐01).
